# Integrating multiomics driven bioengineering and regenerative approaches to improve soil health and productivity in climate adaptive soybean farming on problematic soils

**DOI:** 10.3389/fmicb.2026.1821982

**Published:** 2026-06-30

**Authors:** Fairus Hisanah Hibatullah, Emma Trinurani Sofyan, Anne Nurbaity, Tualar Simarmata

**Affiliations:** 1 Doctoral Student of Agricultural Science Programme, Faculty of Agriculture, Universitas Padjadjaran, Sumedang, Indonesia; 2 Department of Soil Science and Land Resources, Faculty of Agriculture, Universitas Padjadjaran, Sumedang, Indonesia

**Keywords:** climate resilience, multiomics integration, regenerative agriculture, rhizobiome engineering, soil acidity, soil salinity

## Abstract

Multiomics-based bioengineering and regeneration approaches are increasingly recognized as beneficial for restoring soil health and improving sustainable agriculture under climate change. Soil-related abiotic stresses, particularly soil acidity and salinity, continue to be significant production constraints for soybean (*Glycine max* L.) and agroecosystem resilience, particularly in Indonesia’s problematic soils. This study comprehensively reviews integrating multiomics, including genomic, transcriptomic, and rhizomicrobiome-based engineering with regenerative practice to enhance soil biological function, nutrient use efficiencies, and climate-resilient soybean production. A PRISMA-guided systematic review with bibliometric analysis of 2015–2025 publications included 986 articles from ScienceDirect and Scopus, of which 15 were eligible. The research unveils and advocates emerging trends in rhizobiome engineering, multiomics integration, regenerative soil management, and bioameliorant innovations to mitigate abiotic stresses while collaboratively restoring soil functionality. It is concluded that, under acidic and saline soil conditions, the soybean physiological performance for increased stress tolerance was significantly improved by microbial inoculants, nutrient amendments, and CRISPR/Cas9-mediated gene knockout techniques, resulting in an average yield increase of 15%–45% and grain yield exceeding 3.2 t ha^–1^. Additionally, soil pH was raised by 0.5–1.0 units, soil organic carbon increased by 40%, soil biota abundance increased by 50%, and nitrogen-use efficiency increased by 30% through regenerative practices. Despite these developments, long-term field validation, multiomics data integration, and policy support for widespread adoption remain significant obstacles. This review emphasizes climate-smart soybean cultivation on degraded soils by integrating multiomics-based bioengineering with regenerative management approaches. Nevertheless, the limited number of existing studies underscores the need for broader large-scale validation and enhanced data integration.

## 1 Introduction

Integrated multiomics and regenerative approaches are increasingly recognized as transformative strategies for restoring soil health and improving soybean productivity on degraded and stress-prone soils (Khangura et al., 2023; Kumar et al., 2023). By integrating genomics, transcriptomics, metabolomics, and rhizomicrobiome analyses with regenerative practices such as microbial inoculation, organic amendments, and soil organic matter restoration these approaches enable a mechanistic understanding and targeted regulation of plant–microbe–soil interactions under adverse environmental conditions (Yusuf et al., 2025). This framework is particularly critical as soil acidity and salinity intensify as global constraints on sustainable agriculture. Projections indicate that up to 50% of arable land may be affected by salinity by 2050 (Ren et al., 2023b). These stresses are most severe in arid, semi-arid, and coastal regions, where they degrade soil physicochemical properties, suppress biological activity, and substantially reduce crop yields, threatening food security (Huang et al., 2023; Kumar Arora et al., 2020). Soybean, a key source of plant protein and oil, is highly sensitive to acidic and saline conditions, underscoring the urgency of adopting climate-resilient, soil-restorative, and data-driven agricultural strategies (Cheng et al., 2024).

Soybean (*Glycine max* L.) is one of the world’s most economically important legume crops, serving as a major source of plant-based protein, oil, and diverse bioactive compounds (Swallah et al., 2023). Beyond its nutritional and industrial value, soybean plays a critical role in soil fertility improvement through symbiotic nitrogen fixation, positioning it as a key component of sustainable and regenerative agricultural systems (Contador et al., 2020). In Indonesia, soybean is a strategic food crop that underpins national food security, supports smallholder livelihoods, and supplies raw material for staple foods such as tempeh and tofu. Agronomically, soybean functions as an effective soil builder in crop rotations, enhancing soil organic matter and reducing dependence on synthetic nitrogen fertilizers. Despite its importance, soybean productivity is increasingly constrained by soil degradation, particularly acidity and salinity, which together affect nearly 50% of global arable land (Nurbekova et al., 2024).

Soil acidity, prevalent in tropical and subtropical regions, represents a major form of problematic soil that causes toxic accumulation of aluminum (Al^3+^) and manganese (Mn^2+^) while reducing the availability of essential nutrients such as phosphorus, calcium, and magnesium (Liu et al., 2025; Wen et al., 2023). These conditions can suppress soybean root growth by approximately 30%–60% and result in yield losses ranging from 20% to 50% in strongly acidic soils (Shetty and Prakash, 2020; Wen et al., 2023). Conversely, soil salinity another widespread problematic soil constraint induces osmotic stress, ionic toxicity from Na^+^ and Cl^–^, and nutrient imbalance, thereby limiting water uptake and photosynthetic efficiency (Noor et al., 2024; Otie et al., 2021). Moderate to severe salinity has been reported to reduce soybean biomass and grain yield by 40%–80%, depending on salinity intensity and growth stage (Noor et al., 2024; Otie et al., 2021). Collectively, soil acidity and soil salinity disrupt nodulation, rhizosphere microbial diversity, and plant–microbe signaling, rendering soybean particularly vulnerable due to its moderate sensitivity to both stresses (Shetty and Prakash, 2020).

Recent breakthroughs in plant and microbial bioengineering have introduced novel tools to mitigate these soil stresses by modifying plant physiology, enhancing microbial function, and restoring soil ecosystem services (Alharbi et al., 2024; Ren et al., 2023b). Technologies such as CRISPR-Cas gene editing, synthetic biology, and multiomics-guided microbial selection have accelerated the design of climate-resilient soybean varieties and beneficial microbial consortia (Cardi et al., 2023; Li et al., 2021). Unlike conventional breeding, bioengineering enables precise manipulation of genes and metabolic pathways involved in ion transport, antioxidant defenses, and stress signaling, leading to improved adaptation under acidic and saline conditions (Chen X. et al., 2024; Li et al., 2021).

The rhizobiome, the dynamic microbial community associated with plant roots, plays a fundamental role in mediating plant tolerance to soil constraints (Yusuf et al., 2025). Beneficial microbes particularly plant growth-promoting rhizobacteria (PGPR), mycorrhizal fungi, and diazotrophic bacteria enhance nutrient solubilization, root development, and stress resistance (Lin et al., 2025; Tariq et al., 2025; Wen et al., 2023). In acidic soils, phosphate-solubilizing bacteria and acid-tolerant Bradyrhizobium strains improve phosphorus and nitrogen availability (Wen et al., 2023), while in saline soils, halotolerant PGPR help regulate osmolyte accumulation and Na^+^/K^+^ homeostasis (Lin et al., 2025; Liu et al., 2022). These interactions are now being systematically engineered through synthetic microbial consortia (SynComs) that simulate and enhance natural microbiome networks (Tariq et al., 2025; Wang C. et al., 2021).

The advent of multiomics technologies including genomics, transcriptomics, proteomics, and metabolomics has revolutionized our understanding of plant–microbe–soil interactions under stress (Ilangumaran et al., 2022; Jin et al., 2021). Integrating multiomics data allows researchers to identify key genes, metabolites, and microbial pathways responsible for resilience mechanisms in soybean roots exposed to acidity or salinity (Fu et al., 2024). Such insights support rhizobiome bioengineering, where microbial traits are selected or enhanced based on omics-guided performance indicators, enabling the construction of tailored microbial consortia optimized for specific soil conditions (Gonçalves et al., 2023; Liu et al., 2019). Despite the growing body of research on microbial inoculants and soil conditioners, a systematic synthesis of bioengineering strategies targeting soil acidity and salinity in soybean systems remains limited. Therefore, this review assesses recent advances and emerging trends in multiomics-based bioengineering and regenerative approaches for mitigating soil acidity and salinity, with the aim of restoring soil health and improving soybean productivity in climate-adapted farming systems. It also addresses key constraints, including the high cost of multiomics technologies, limited field-scale validation, and difficulties in translating complex datasets into practical management strategies. Major research gaps are highlighted, particularly the limited understanding of multi-stress interactions, region-specific soil–microbiome responses, and the long-term stability of regenerative interventions. Future perspectives emphasize the development of scalable, cost-effective multiomics tools, integrative modeling frameworks, and supportive policies to enable resilient soybean production on degraded soils.

## 2 Methodology

### 2.1 Literature search strategy

The literature was searched using systematic literature review and bibliometric analysis methods, using the Scopus and ScienceDirect databases (Van Eck and Waltman, 2010; Page et al., 2021). The search terms used were by combining “soybean,” “bioengineering,” “rhizobiome,” “soil acidity,” “salinity,” “multiomics,” and “bioameliorant” like in Table 1. Amounts of papers published by various categories, including journals, year, and nation, were included in the data that was retrieved. The search result data, as in Table 1, was checked for duplicates and then removed using Mendeley Reference Manager 2.115.0, leaving 15 articles for analysis.

**TABLE 1:** Literature search method used to retrieve relevant research articles.

Search strategy	Scopus	ScienceDirect
((“soybean” OR “*Glycine max*”) AND (“soil acidity” OR “acidic soil” OR “low pH soil”))	100	259
(“soybean” OR “*Glycine max*”) AND (“bioengineered rhizobacteria” OR “PGPR inoculant” OR “engineered microbes”))	3	5
(“soybean” OR “legume crop”) AND (“CRISPR” OR “gene editing”) AND (“abiotic stress tolerance” OR “climate resilience”))	3	40
(“genetic engineering” OR “transgenic soybean” OR “PGPR”) AND (“acidic soil” OR “saline soil” OR “climate adaptation”))	60	130
((“soybean” OR “bioengineering”) AND (“multiomics” OR “metabolomics” OR “genomics” OR “proteomics”) AND (“saline soil” OR “acid soil” OR “degraded soil”))	2	112
(“soybean” OR “*Glycine max*”) AND (“bioengineering” OR “biofertilizer”) AND (“climate resilience” OR “sustainable agriculture” OR “stress tolerance”))	8	186
(“soybean” OR “*Glycine max*”) AND (“multiomics” OR “metabolomics” OR “genomics” OR “proteomics”) AND (“bioameliorant” OR “biochar” OR “compost”))	4	74
Total	180	806
Total articles		986

### 2.2 Inclusion and exclusion criteria

The retrieved documents were screened according to predefined selection criteria (Table 2). Bibliographic records were exported in RIS format and analyzed using VOSviewer version 1.6.19 to perform an in-depth bibliometric assessment. The analysis examined co-occurrence relationships among terms, keywords, and citation patterns. A minimum occurrence threshold of 12 was applied to ensure that only frequently occurring and thematically relevant terms were included, thereby reducing disruption and improving the reliability of the bibliometric network, yielding 462 relevant terms. From these, the top 60% based on relevance scores were selected, resulting in 277 terms for visualization. Prior to mapping, the dataset was refined by excluding non-informative or potentially confounding terms such as “fish,” “rotation,” “depth,” “plot,” “member,” “replicate,” and “heat” to improve analytical clarity and reduce bias.

**TABLE 2:** Screening criteria used for screening documents obtained from database.

Criteria	Inclusion	Exclusion
Relevance topics	Journal with focus on soybean under acidic or saline soil conditions. Use of bioengineering, microbial inoculants, or omics-based soil health strategies.	Journal without core focus on bioengineering on soybean under acidic or saline soil conditions.
Publication year	2015–2025	Years before 2015
Publication type	Research article	Books chapter, encyclopedia, news, conference abstracts
Language	English	All other language
Databases	Scopus and ScienceDirect	Articles that are not indexed by Scopus

### 2.3 Data extraction and synthesis

**FIGURE 1:**
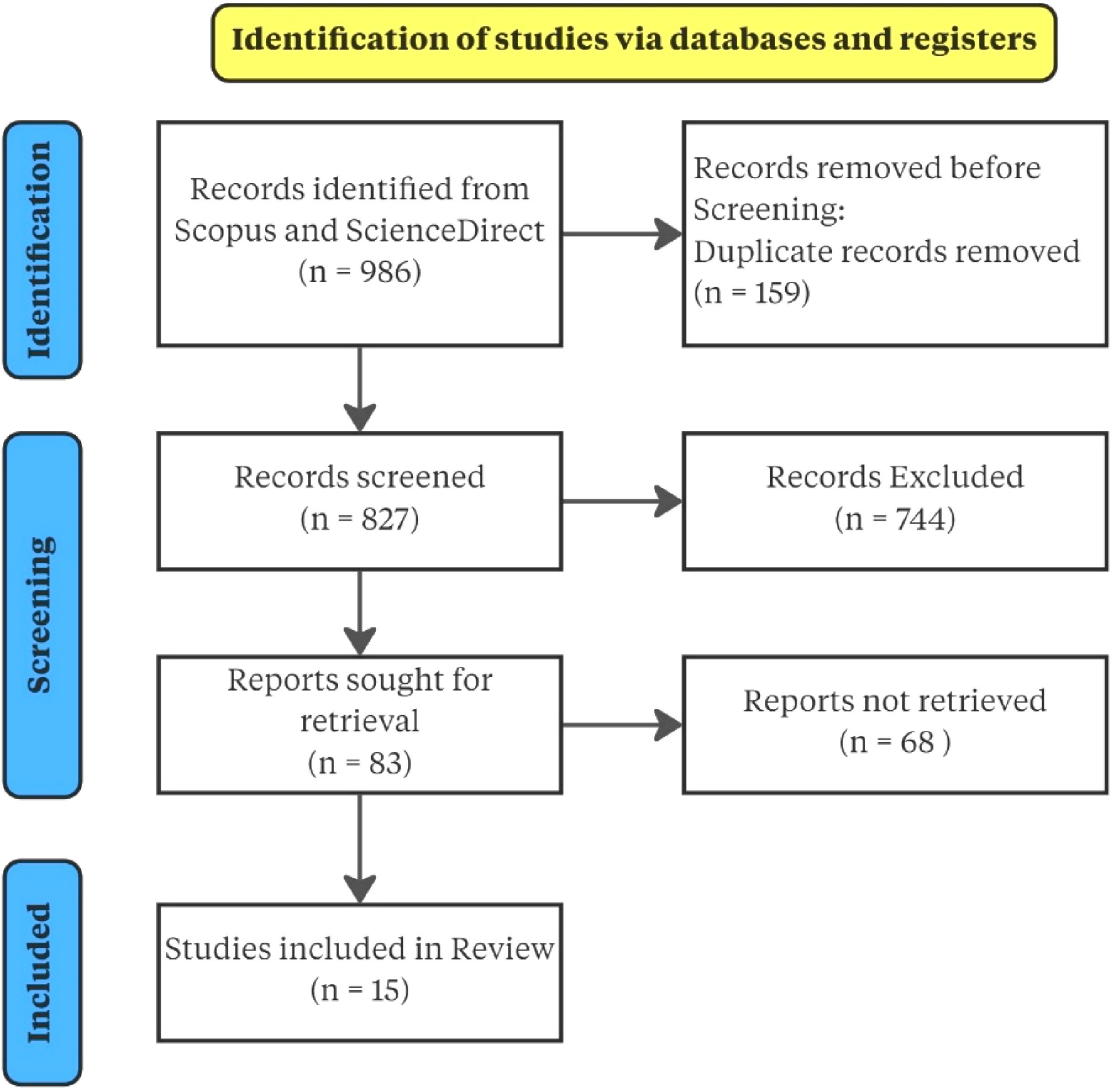
The procedure for identifying, screening, and selecting articles for the systematic literature review followed the PRISMA flow framework.

This review was performed following the PRISMA framework, as depicted in Figure 1, to ensure systematic and transparent methodology (Page et al., 2021). An extensive literature search was performed using the Scopus and ScienceDirect databases. Keywords included “Bioengineering,” “Salinity,” “Tolerance,” “Acid,” “Soil,” and “Soybean.” Studies were included if they focused on rhizomicrobiome engineering, were relevant to degraded soils, and demonstrated potential applications in global agricultural systems. All identified articles were screened for relevance, and data were extracted and synthesized to summarize current advancements and their implications. Extracted data included research objectives, microbial strains, bioengineering approaches, and reported outcomes. The final selection of 15 articles reflects strict filtering to ensure high relevance to multiomics-based bioengineering in soybean under acidic and saline soils.

### 2.4 Bibliometric framework and analytical scope

A conceptual bibliometric analysis identified research trends, collaboration networks, and thematic evolution (Donthu et al., 2021). The focus was on:

1. Keyword co-occurrence – dominant research themes.

2. Authorship collaboration – research hubs and partnerships.

3. Citation trends – evolution from microbial inoculants to multiomics approaches.

## 3 Results and discussion

### 3.1 Bibliometric analysis

#### 3.1.1 Relevant source

**FIGURE 2:**
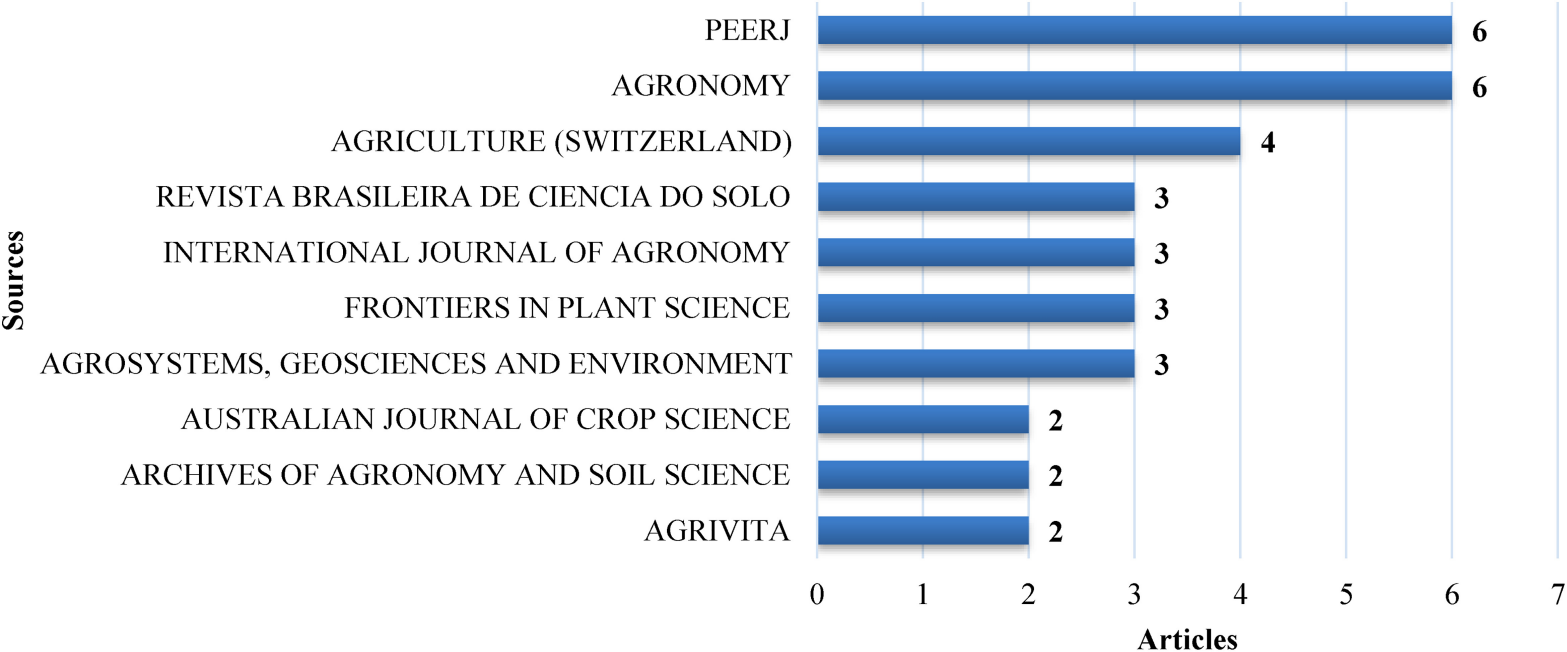
Most relevant sources in the literature on multiomics bioengineering for climate-resilient soybean on problematic soils.

The most relevant journals publishing research on integrating multiomics-driven bioengineering and regenerative approaches to improve soil health and productivity in climate-adaptive soybean farming on problematic soils (Figure 2) are led by PEERJ and Agronomy, each contributing six articles. This dominance reflects their strong focus on interdisciplinary studies that connect molecular-scale insights with agronomic and regenerative practices. Agriculture (Switzerland) follows with four articles, highlighting the growing emphasis on sustainability and climate resilience in crop production systems.

A second group of journals including Revista Brasileira de Ciência do Solo, International Journal of Agronomy, Frontiers in Plant Science, and Agrosystems, Geosciences and Environment, each contributes three articles, underscoring the importance of soil processes, plant–microbe interactions, and systems-level analyses. Additional contributions from the Australian Journal of Crop Science, Archives of Agronomy and Soil Science, and Agrivita reflect applied and regional research perspectives. This distribution indicates that the field is strongly anchored in agronomy and soil science, demonstrating the translation of advanced multiomics approaches into practical, regenerative, and climate-smart soybean farming strategies.

#### 3.1.2 Relevant author

**FIGURE 3:**
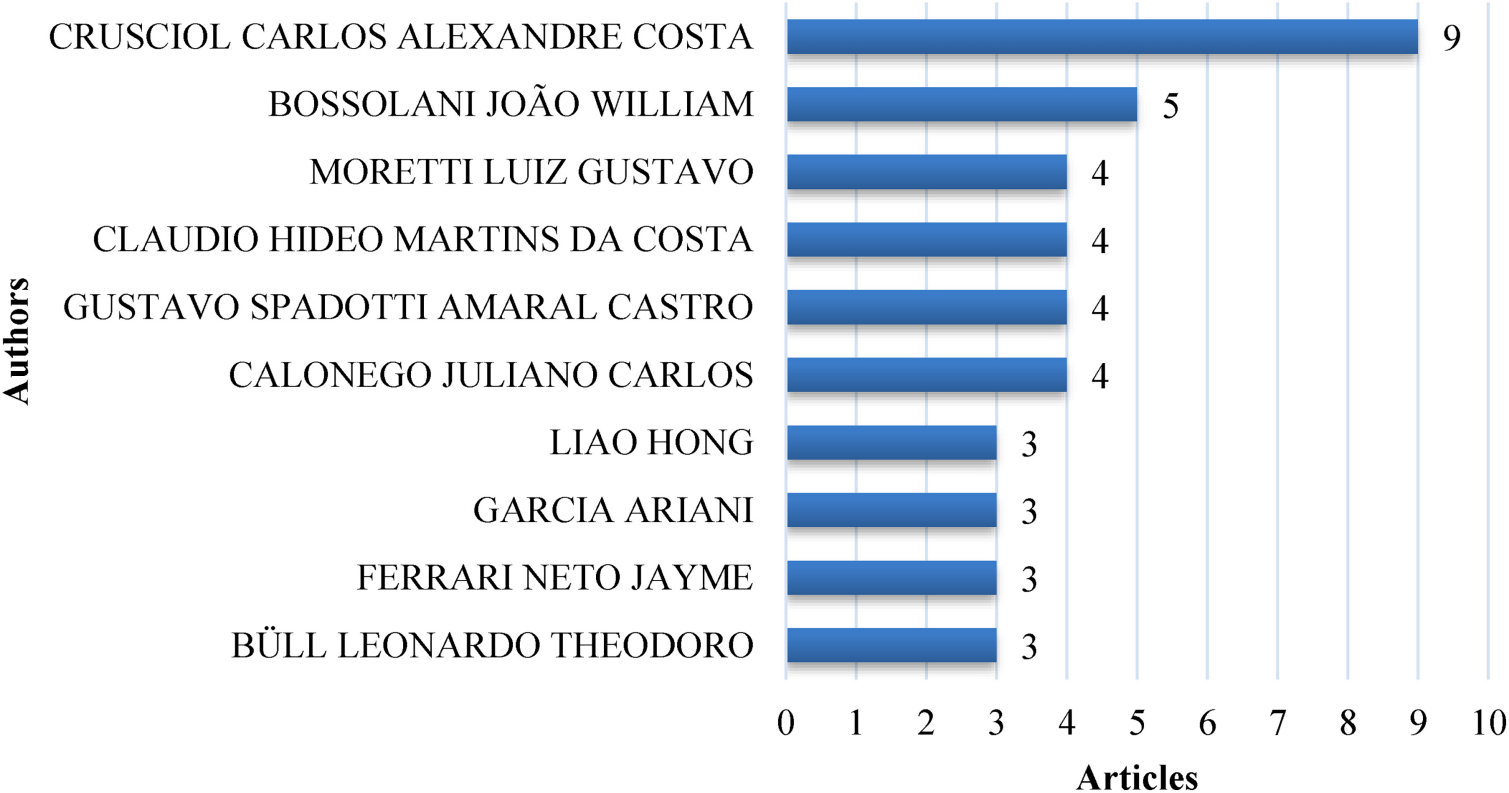
Most relevant authors contributing to research on multiomics bioengineering for climate-resilient soybean on problematic soils.

The most relevant authors contributing to the literature on multiomics bioengineering for climate-resilient soybean production on problematic soils (Figure 3) highlight key contributors and research leadership within this field. “Crusciol Carlos Alexandre Costa” emerges as the most prolific author, with nine publications, reflecting a sustained and influential role in advancing soil–plant system research that integrates soil management, crop physiology, and stress resilience. “Bossolani João William” follows with five articles, while “Moretti Luiz Gustavo,” “Costa Claudio Hideo Martins,” “Castro Gustavo Spadotti Amaral,” and “Calonego Juliano Carlos” each contribute four publications, indicating strong collaborative research networks. Additional contributions from “Liao Hong,” “Garcia Ariani,” “Ferrari Neto Jayme,” and “Büll Leonardo Theodoro,” each with three articles, further demonstrate the collective and interdisciplinary nature of this research area. Overall, the figure shows a concentration of expertise rooted in agronomy and soil science, emphasizing the importance of sustained, team-based research efforts in integrating multiomics approaches with practical strategies to enhance soybean resilience, soil health, and productivity under climate stress conditions.

#### 3.1.3 Relevant affiliation

**FIGURE 4:**
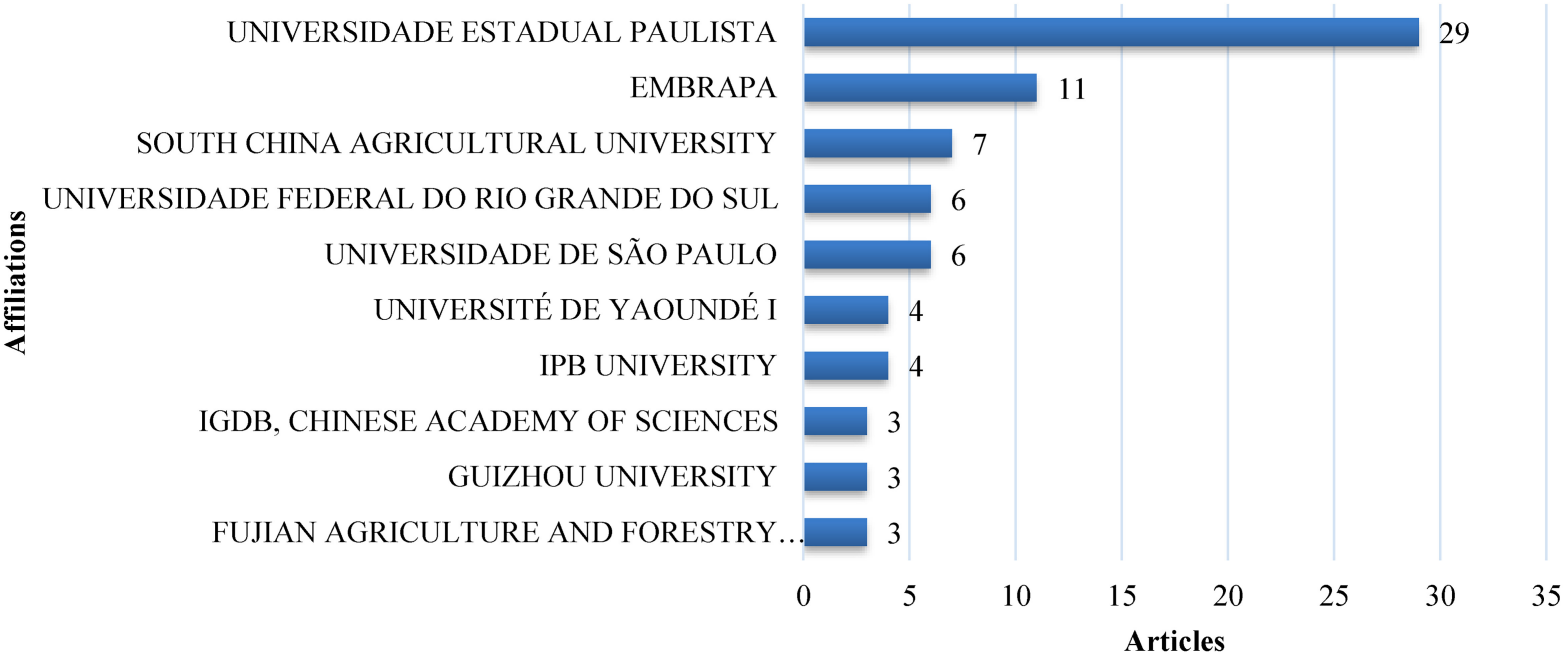
Most relevant institutional affiliations in multiomics bioengineering for climate-resilient soybean on problematic soils.

The most institutional relevant affiliations contributing to the literature on multiomics bioengineering for climate-resilient soybean production on problematic soils (Figure 4), revealing clear institutional leadership and geographic concentration. Universidade Estadual Paulista (UNESP) in São Paulo, Brazil dominates the field with 29 publications, underscoring its central role in advancing integrative research on soil management, crop physiology, and multiomics-driven innovation. The Empresa Brasileira de Pesquisa Agropecuária (EMBRAPA), Brazil follows with 11 articles, reflecting Brazil’s strong national research capacity in soybean systems and climate-smart agriculture.

International contributions are led by South China Agricultural University in Guangzhou, Guangdong, China (seven articles), highlighting Asia’s growing engagement in multiomics and stress-resilient crop research. Brazilian institutions such as the Universidade Federal do Rio Grande do Sul in Porto Alegre, Brazil and the Universidade de São Paulo in São Paulo, Brazil (six articles each) further reinforce the prominence of South America in this domain. Additional participation from African and Asian universities, including Université de Yaoundé I in Yaoundé, the capital city of Cameroon, IPB University in Bogor, Indonesia, and Guizhou University in Guiyang, Guizhou, China illustrates expanding global collaboration. Overall, the figure emphasizes that sustained institutional investment and international partnerships are driving progress in multiomics-based, climate-resilient soybean research on challenging soils.

#### 3.1.4 Word cloud

**FIGURE 5:**
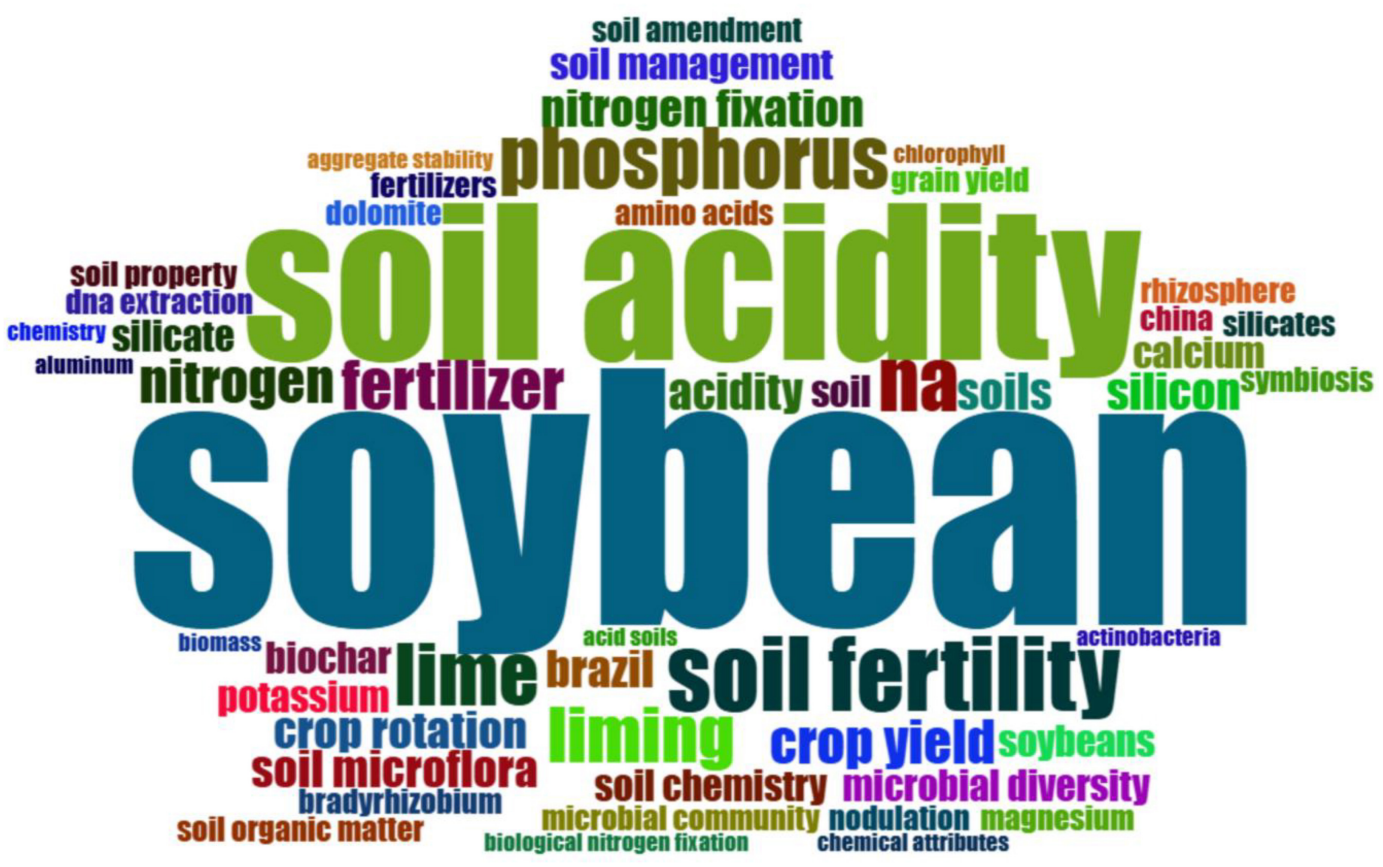
Word cloud of the most frequent terms in multiomics bioengineering for climate-resilient soybean on problematic soils publications.

The word cloud analysis (Figure 5) illustrates the most frequent terms appearing in publications on multiomics bioengineering for climate-resilient soybean production on problematic soils, offering insight into dominant research themes and conceptual priorities. The prominence of “soybean” and “*Glycine max*” confirms the central crop focus, while “soil acidity” stands out as a major constraint shaping research efforts. Related terms such as “lime,” “phosphorus,” “nitrogen,” and “soil fertility” emphasize nutrient limitations and chemical imbalances commonly associated with degraded or marginal soils. Biological and ecological dimensions are reflected in recurring terms like “microbial diversity,” “rhizosphere,” “symbiosis,” and “nitrogen fixation,” highlighting the importance of plant–microbe interactions and microbiome-based interventions within multiomics approaches. Management-oriented concepts including “zero tillage,” “crop rotation,” “biochar,” and “soil amendment” point to the integration of regenerative practices with molecular and systems-level insights. Overall, the figure reveals a strong convergence of soil chemistry, microbial ecology, and sustainable management strategies aimed at improving soybean resilience and productivity under climate stress conditions.

### 3.2 Bibliometric overview of research trends by VosViewer

The bibliometric analysis revealed a rapid expansion in research linking bioengineering, rhizobiome management, and multiomics technologies for stress-prone soybean farming between 2015 and 2025. Annual publication trends indicated a gradual rise during 2015–2015, followed by a significant surge from 2018 onwards, coinciding with the global advancement of multiomics integration and synthetic microbial community (SynCom) applications in agricultural biotechnology (Donthu et al., 2021). The bibliometric analysis provides valuable insights into the intellectual landscape and evolving research priorities associated with bioengineering strategies for enhancing soybean performance in acid and saline affected soils. The VOSviewer network and overlay visualizations reveal clear thematic structures and emerging innovation pathways within the global research community.

**FIGURE 6:**
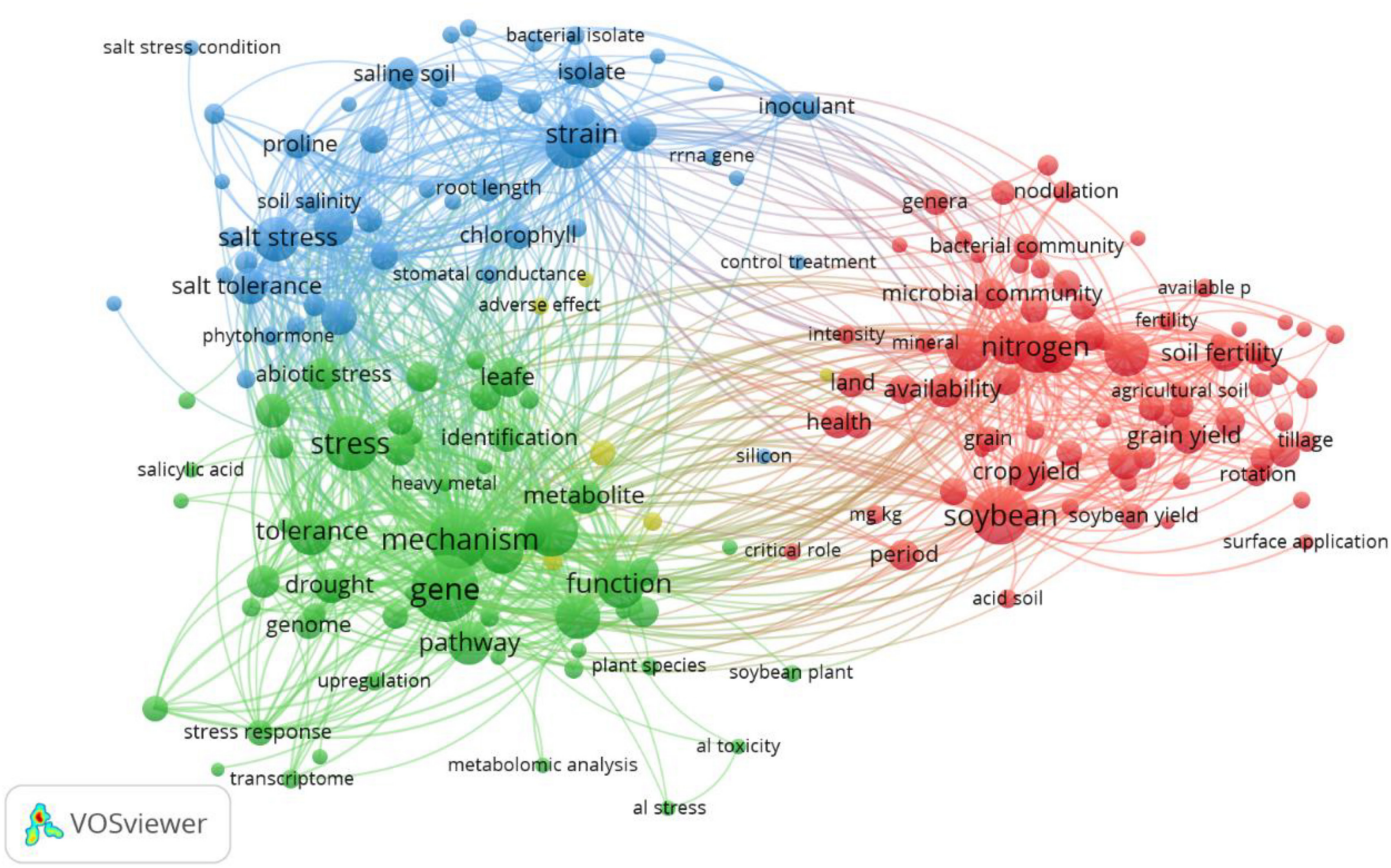
A network visualization of the co-occurrence mapping of pertinent keywords related to research on innovative multiomics bioengineering approaches to address soil acidity and salinity for climate-resilient soybean farming.

The *network visualization* (Figure 6) demonstrates a highly interconnected research ecosystem, where several major clusters converge around plant physiology, soil microbiology, and biotechnological approaches. Prominent keywords such as *soybean*, *Bradyrhizobium*, *salinity stress*, *acid soil*, *nitrogen fixation*, *phosphorus solubilization*, and *plant growth-promoting rhizobacteria (PGPR)* indicate sustained interest in leveraging beneficial microbial interactions to enhance plant tolerance and yield under abiotic stress. The dense clustering further highlights the integration of classical agronomy with microbial ecology and biofertilizer development, reflecting a paradigm shift from purely chemical amendments toward biologically driven soil fertility restoration.

**FIGURE 7:**
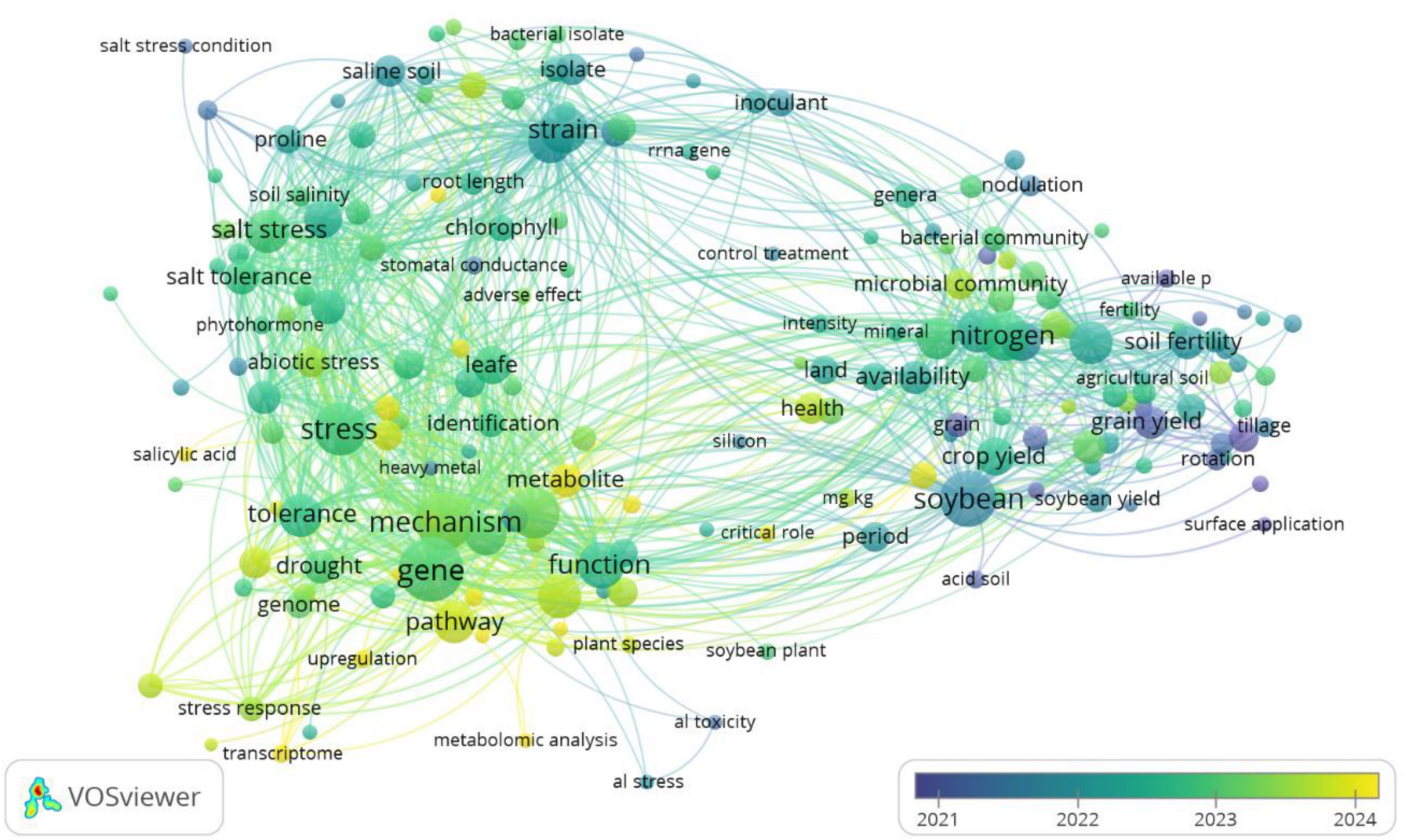
An overlay visualization of the publication year mapping related to research on innovative multiomics bioengineering approaches to address soil acidity and salinity for climate-resilient soybean farming.

The *overlay visualization* (Figure 7) indicates a temporal shift in scientific focus. Earlier studies were dominated by foundational work on soil acidity, salinity tolerance, and legume–rhizobium symbiosis. More recent research, particularly between 2019 and 2024, reflects the emergence of advanced biotechnological tools. Keywords such as *multi-omics*, *synthetic microbial consortia*, *nanobiotechnology*, *microencapsulation*, and *biopolymer carriers* signal increasing attention to precision engineering of microbial delivery systems and plant–microbe interactions. This progression suggests accelerating interest in controlled microbial deployment and nanoscale bio-stimulant technologies to improve stress adaptation and nutrient use efficiency.

The growing emphasis on these modern techniques is consistent with global efforts to transition toward sustainable and climate-resilient agroecosystems. Advancements in metagenomics, transcriptomics, and metabolomics are driving deeper understanding of rhizosphere microbiomes and stress-response pathways, enabling targeted selection and engineering of microbial inoculants. Additionally, the increased presence of terms related to sustainability, soil health, and climate-smart agriculture highlights the alignment of soybean microbial bioengineering research with broader international goals for ecological intensification and reduced dependence on agrochemicals.

Taken together, the bibliometric evidence underscores a robust and rapidly evolving field, driven by the convergence of plant science, microbial biotechnology, and nanotechnology. The transition from conventional soil improvement strategies toward precision microbiome engineering represents a critical innovation trajectory with significant implications for soybean production in degraded and stress-prone soils. These trends reinforce the need for continued interdisciplinary research efforts and the development of scalable microbial technologies to enhance crop resilience in the context of global climate challenges.

### 3.3 Soil acidity and salinity constraints in soybean production

Soil acidity and salinity are major abiotic stresses limiting soybean productivity worldwide. Acidic soils, which cover a significant portion of arable land, restrict nutrient availability especially phosphorus and increase aluminum toxicity, both of which hinder soybean growth and yield (Ayanso et al., 2025). Salinity, exacerbated by climate change and poor irrigation practices, leads to osmotic stress and ion toxicity, resulting in substantial reductions in plant height, pod number, seed yield, and overall plant health (Osman et al., 2021; Ren et al., 2023a). These constraints necessitate the development of tolerant cultivars and effective soil management strategies to sustain soybean production in affected regions (Ayanso et al., 2025).

Acidic soils restrict root elongation, reduce rhizobial survival, and immobilize essential nutrients such as phosphorus (P), calcium (Ca), and magnesium (Mg), while increasing toxic ions like aluminum (Al^3+^) and manganese (Mn^2+^) (Bossolani et al., 2021; Rahman et al., 2018). Conversely, salinity disrupts osmotic balance and induces ion toxicity, leading to impaired photosynthetic performance and oxidative stress (Farouk and Al-Huqail, 2022). The co-occurrence of acidity and salinity, often exacerbated by climate extremes and poor soil management, significantly compromises soil health and biological fertility (Tarolli et al., 2024).

Traditional chemical approaches such as liming or gypsum application offer temporary relief but often fail to restore the intricate biological and biochemical processes essential for sustainable soil productivity. Therefore, bioengineering strategies that harness microbial resilience and natural soil buffering mechanisms are critical for achieving long-term ecological restoration and climate-resilient soybean production (de Souza et al., 2025).

### 3.4 Microbial bioengineering for soil rehabilitation and productivity of soybean

Microbial bioengineering, including the use of plant growth-promoting microbes (PGPMs), microbial consortia, and targeted inoculants, has emerged as a promising approach to rehabilitate degraded soils and enhance soybean productivity. Inoculation with beneficial microbes improves nutrient cycling, stress tolerance, and disease resistance, leading to increased yield and soil health, even under saline or acidic conditions (Jie and Zhang, 2025; Travençoli Rossetim et al., 2025). The combined application of PGPMs and soil amendments (e.g., biochar, lime, gypsum) further optimizes soil properties, microbial diversity, and plant resilience.

Plant Growth-Promoting Rhizobacteria (PGPR), including *Bradyrhizobium japonicum, Bacillus subtilis*, and *Pseudomonas fluorescens*, have shown capacity to neutralize rhizosphere acidity through organic acid metabolism and ammonia release (Hasan et al., 2024). Meanwhile, halotolerant microbes such as *Halomonas* and *Azospirillum* spp. mitigate salt toxicity by producing osmoprotectants (proline, trehalose) and exopolysaccharides that improve soil structure and moisture retaining (Kumar et al., 2024). This biological reinforcement promotes root growth, nodulation, and N_2_ fixation efficiency essential attributes for climate-resilient soybean farming (Htwe et al., 2019; Kumar et al., 2020). As shown in Table 3, most studies consistently report improvements in plant growth, physiological traits, and stress tolerance, although the magnitude of response varies across experimental conditions.

**TABLE 3:** Summary of reviewed studies on bioengineering approaches to soil acidity and salinity management in soybean farming.

Problematic soil (acidity/ salinity)	Multiomics and bioengineering approach	Soil and plant responses	References
Soil salinity	*Ensifer sesbaniae* DY22 as (PGPR) inoculation	- DY22 inoculation of soybean seeds resulted in better root and stem growth under saline stress or normal conditions - Soybean seeds inoculated with DY22 showed a significant increase in total fresh and dry weight of seedlings compared to those without inoculation, under saline and normal conditions - The chlorophyll content of DY22-inoculated soybeans was significantly higher than that of salt-treated plants. - Soluble sugar and proline concentrations rose when DY22 was injected into salt-stressed soybean seedlings	Sui et al., 2024
Soil salinity	Application of *Azotobacter chroococcum* (Az) and zinc sulfate (Zn)	- Increases in chlorophyll content (a, b, and a+b), carotenoids, RWC, Fv/Fm, MSI, anthocyanins, osmolytes, N, K^+^, P, and K^+^/Na^+^ ratio occurred as a result of Zn application and inoculation with Az. - There was a decrease in CAT, SOD, POX, and APX activity, as well as MDA and Na^+^ content. - The salinity x nutrition interaction effect had a major effect on the ion contents of soybean roots and shoots.	Yaghoubian et al., 2021
Soil salinity	Corn steep liquor fermentate with *Aspergillus oryzae* YY-21 as biostimulant (CSLF)	Soil restoration - There was a significant change in the microbial community structure in the group treated with CSLF, with Gaiellales, 67–14, KD4–96, MB-A2–108, and Gemmatimonadaceae having higher relative abundances, with significant differences between groups. - In the CSLF treatment group, the soil pH value was significantly lower when compared to the CK and CF groups. - Following the application of either CF or CSLF, the microbial community changed, with the CSLF group being more aggregated. Plant response - Organic matter, short peptides, and nitrogen from amino acids all rose by 6.38%, 9.87%, and 17.41%, respectively, in CSLF. - Aspartic acid, glutamic acid, and arginine levels in CSLF increased by 50%, 39%, and 1.22%, respectively. - CSLF application increases plant height, stem diameter, leaf area, fresh weight, and dry weight of soybean seedlings.	Jiang et al., 2025
Soil salinity	CRISPR/Cas9-generated Gm-miR396a–edited lines (miR396a-GEs)	- Compared to control plants, Gm-miR396a gene-edited lines (miR396a-GEs), which were produced using CRISPR/Cas9, showed more branches, higher grain yields, and increased resistance to salinity. - Hormone-regulating biological processes were markedly enriched in the transcripts of lines with altered miR396a-GE abundance. - Developmental deficits such as dwarfism, aberrant inflorescences and flowers, smaller and fewer seeds, and short leaves with larger and more frequent stomata were caused by overexpression of the Gm-miR396a precursor (pre-miR396a-OE). - These findings demonstrated that, under typical growing conditions, modifying Gm-miR396a increased grain production by an average of 16%.	Chen X. et al., 2024
Soil acidity	Phosphorus application with rhizobium inoculant	- Plant height, the number of pods per plant, and the yield of grain and husk all varied significantly as a result of the provided stimulus. - Phosphorus and inoculants are applied at a substantially higher rate (19%). enhanced soybean production and yield-related characteristics.	Zerihun et al., 2015
Soil acidity	Application of phosphate solubilizing bacteria	- In comparison to the control (without dolomite and P-solubilizing bacteria), isolate Lampung-2 enhanced seed yield by 18% and by 40% when paired with dolomite equivalent to 800 kg ha^–1^. - The application of Lampung-2 + 200 kg ha^–1^ SP36 resulted in the best yield and a 45% increase in yield compared to the uninoculated controls with 0 kg ha^–1^ SP36. - The field experiment’s findings demonstrated that Lampung-2 could boost production by 27% (equal to seed yield obtained from 200 kg ha^–1^ SP36) at a fertilization dose of 100 kg ha^–1^ SP36, followed by Lampung-1 with 18.5% over the control treatment.	Widodo et al., 2022
Soil acidity	Application of local soybean rhizobia such as SRU11, SRU25, SRU26, SRU27, SRU31, SRU36, and SRU70	- The highest nodule number, nodule wet weight, nodule dry weight, shoot height, shoot dry weight, shoot wet weight, and shoot length were recorded as 38 ± 1 (SRU66), 1.71 ± 0.1 g/plant (SRU66), 0.63 ± 0.49 g/plant (SRU19), 36.7 ± 1 cm/plant (SRU36), 1.41 g/plant (SRU25), 10.8 ± 1 g/plant (SRU1), and 72.0 ± 1 cm/plant (SRU1).	Gelan et al., 2025
Soil acidity	Biofertilizer and inorganic fertilizers	- The interactions between biofertilizer and inorganic fertilizers at both locations and years had a highly significant (*P* < 0.01) impact on the number of effective nodules per plant, leaf area index, and grain production. - Therefore, SB12+MAR1495 + NPSB at Assosa produced the highest grain yield (2621.67 kg), while SB12+NPS at Bambassi produced the highest grain yield (2460.20 kg). Therefore, for increased soybean grain production, (SB12+MAR1495) + NPSB and SB12+NPS are advised.	(Abeje et al., 2022)
Soil salinity	Transgene-free gmaitr36 double and gmaitr23456 quintuple mutants developed via CRISPR/Cas9 gene editing	- When GmAITR genes are mutated by CRISPR/Cas9 genome editing, soybeans become more tolerant of salinity. - The *gmaitr* mutant seedlings produced longer roots and shoots than the Wm82 wild type seedlings, indicating that they were more tolerant of salt treatment.	Wang T. et al., 2021
Soil acidity	Application of rhizobia inoculants (*Bradyrhizobium japonicum* strain 532C and *Bradyrhizobium diazoefficiens* strain USDA110)	- In moderately acidic soils, soybean inoculation effectively increases nodulation and N fixation. - Shoot dry weight increased by 81.09% after Legume fix inoculation and by 17.08% after Biofix inoculation.	Bakari et al., 2020
Soil acidity	Combine of *Bradyrhizobium* inoculation, lime and phosphorus fertilizer	- The TAL379 inoculation significantly increased the number of primary branches (6.7), harvest index (41%), and grain output (3228 kg ha^–1^). - Days to physiological maturity and the number of pods per plant were significantly impacted by the combination of P and *Bradyrhizobium* inoculation.	Dabesa and Tana, 2021
Soil acidity	inoculation with *Rhizobium* strains	- After being inoculated with strains S1 (2.93 ± 0.06% vs. 2.53 ± 0.12%) and S2 (3.34 ± 0.05% vs. 2.53 ± 0.12%), the total nitrogen content of the var. TGX 1910 14F rose. - The root fresh weight of TGX 1910 14F showed a similar pattern. Plant height, above-ground biomass, and fresh and dry root weights were increased in Maksoy 4N treated with strain S1. - The inoculated seeds had the greatest number of nodules, and strain S2 was the most successful of all the soybean cultivars. - In terms of yield, strain S1 was most productive for TGX 1910 14F (169.66 ± 75.56 seeds per plant, or 1.35 ± 0.60 tons/ha) and Maksoy 4N (106.0 ± 2.64 seeds per plant, or 0.84 ± 0.02 tons/ha), whereas strain S2 was most productive for TGX 1835 10E (174.33 ± 0.34 tons/ha).	Manet et al., 2025
Soil acidity	Application of rhizobial inoculants	- Soybean production was increased by inoculation with either the commercial strain or one of two locally generated isolates that were created as inoculants.	Muleta et al., 2017
Soil acidity	Arbuscular mycorrhizal fungi (AMF) and P-fertilization application	Soil restoration - The composition of the bacterial community was unaffected by the AMF inoculation and P-fertilization treatments, but the phylum-level composition of the fungal community was unexpectedly impacted. - For the greater P-efficiency soybean PT6, the AMF inoculation boosted Zygomycota and Cercozoa abundance in SS. Plant response - AMF inoculation dramatically boosted the plant biomass of greater P-efficiency transgenic soybean PT6 by 46.74%–65.22%.	Wen et al., 2024
Soil acidity	*B. japonicum* inoculant and rice husk biochar + compost or phosphorus fertilizer	- Plants growing in soils treated with B (+26), B + CM (+53), B + RP (+39), and B + TSP (+40) had more nodules due to *B. japonicum*. - When biochar treatment was paired with compost or P sources, there was a notable rise in pH, cation exchange capacity, and P availability, which led to an increase in grain output.	Asirifi et al., 2025

### 3.5 Bioengineering and regenerative approaches in addressing abiotic stress and improving soybean productivity

Abiotic stresses such as soil acidity, salinity, and nutrient imbalance are among the most significant constraints limiting soybean (*Glycine max* L.) productivity, soil fertility, and agroecosystem sustainability in tropical and subtropical regions (Ayanso et al., 2025; Sankhala et al., 2024). Recent advances in bioengineering and regenerative approaches offer integrated solutions to overcome these challenges through synergistic plant–microbe–soil interactions, bioremediation, and soil function restoration (Moretti et al., 2020; Singh et al., 2023).

Recent studies demonstrate substantial progress in applying multiomics-informed bioengineering and regenerative strategies to mitigate soil acidity and salinity constraints in soybean farming, with clear quantitative benefits. Under saline conditions, plant growth–promoting rhizobacteria such as *Ensifer sesbaniae* DY22 significantly increased shoot and root biomass, fresh and dry weights, chlorophyll content, and osmolyte accumulation, enabling soybean seedlings to perform better under both stress and non-stress conditions (Sui et al., 2024). Similarly, combined application of zinc sulfate and *Azotobacter chroococcum* improved chlorophyll fluorescence (Fv/Fm), membrane stability, K^+^/Na^+^ ratios, and antioxidant enzyme activities, while reducing Na^+^ and malondialdehyde levels (Yaghoubian et al., 2021). Gene-editing approaches further demonstrated strong yield impacts, with CRISPR/Cas9-edited *Gm-miR396a* lines achieving an average 16% grain yield increase under normal conditions and enhanced salinity tolerance (Chen F. et al., 2024), while *gmaitr* mutants showed longer roots and shoots under salt stress (Wang T. et al., 2021).

In acidic soils, bioengineering strategies consistently improved productivity. Rhizobium inoculation combined with phosphorus increased yields by 19% (Zerihun et al., 2015), while phosphate-solubilizing bacteria with dolomite or SP36 fertilizer achieved yield increases of 27%–45% (Widodo et al., 2022). Integrated applications of biochar, lime, rhizobia, and fertilizers raised soil pH, cation exchange capacity, nodulation, and grain yields up to 3,228 kg ha^–1^ (Dabesa and Tana, 2021; Asirifi et al., 2025). Collectively, these quantitative outcomes demonstrate that integrated microbial, genetic, and soil-amendment approaches can substantially enhance soybean resilience and productivity on problematic acidic and saline soils.

The results summarized in Table 4 demonstrate that multiomics bioengineering and regenerative approaches provide robust and complementary solutions for mitigating abiotic stresses and improving soybean productivity under problematic soil conditions. Rhizobiome engineering using synthetic microbial consortia (SynComs) showed strong performance across acidic and saline soils by precisely designing microbial functions related to nitrogen fixation, phosphorus solubilization, and stress signaling. These targeted interventions translated into substantial agronomic gains, with soybean yields increasing by 25%–35% and nitrogen-use efficiency improving by approximately 30%, highlighting the effectiveness of microbiome-level manipulation for nutrient optimization and stress resilience (Nadarajah and Abdul Rahman, 2023).

**TABLE 4:** Performance of multiomics bioengineering and regenerative approaches in addressing abiotic stress and improving soybean productivity.

Problematic soil (salinity/acidity)	Approach	Mode of action	Quantitative improvement
Soil acidity, salinity	Rhizobiome engineering (SynComs)	Precision microbial consortia design for N-fixation, P-solubilization, and stress signaling	Yield increased by 25%–35%, accompanied by an approximately 30% improvement in N- use efficiency (Nadarajah and Abdul Rahman, 2023).
Acidity, salinity, drought	Multiomics-guided bioengineering	Integration of genomics, transcriptomics, and metabolomics for stress-responsive trait enhancement	Yield increased by 20%–30% (Adeleke et al., 2024).
Soil acidity, salinity	Bioameliorant application (biochar, compost)	Soil pH correction, C-sequestration, microbial habitat improvement	Soil pH increased by 0.5–1.0 units, while soil organic carbon content rose by approximately 40% (Lehmann and Joseph, 2015).
Multi-stress conditions	Regenerative soil management	Crop residue recycling, green manure, and organic amendments	Crop yield increased by 15%–25%, accompanied by an approximately 50% increase in soil biota abundance (Singh et al., 2023)

Multiomics-guided bioengineering further enhanced soybean performance under combined acidity, salinity, and drought stress by integrating genomics, transcriptomics, and metabolomics to identify and regulate stress-responsive traits. Yield improvements of 20%–30% reported in these studies underscore the value of systems-level approaches for decoding complex stress tolerance mechanisms (Adeleke et al., 2024). In parallel, regenerative bioameliorant applications such as biochar and compost played a critical role in restoring soil function. These practices corrected soil pH by 0.5–1.0 units and increased soil organic carbon by about 40%, creating a more favorable habitat for microbial activity and root development (Lehmann and Joseph, 2015).

Finally, regenerative soil management practices, including crop residue recycling and organic amendments, delivered broader ecosystem benefits by increasing yields by 15%–25% while enhancing soil biota abundance by approximately 50% (Singh et al., 2023). Collectively, these findings emphasize that integrating multiomics-driven bioengineering with regenerative soil management offers synergistic, scalable, and climate-resilient pathways for sustainable soybean production under abiotic stress conditions.

### 3.6 Omics-driven insights for rhizobiome engineering

The advent of multi-omics technologies including metagenomics, transcriptomics, and metabolomics has transformed our ability to decipher the functional ecology of rhizosphere microbiomes (Yusuf et al., 2025). Through these approaches, specific gene clusters related to stress signaling, ion transport, and hormone biosynthesis can be identified and targeted to enhance soybean adaptability (Pruthi et al., 2025).

Advances in omics technologies metagenomics, metabolomics, and transcriptomics have revolutionized the understanding of rhizosphere microbiomes. These tools reveal the complex interactions between soybean roots and microbial communities, identifying key taxa and functional genes involved in nutrient cycling, stress adaptation, and plant growth promotion (Gai et al., 2025; Yusuf et al., 2025). Omics-driven approaches enable the design of synthetic microbial communities (SynComs) and precision interventions to engineer the rhizobiome for enhanced crop performance.

For instance, metagenomic profiling of saline and pH-variable soils reveals pronounced shifts in microbial community structure and functional gene repertoires along water–salt and pH gradients (Yang et al., 2022; Zhang et al., 2024). Metabolomic studies complement these findings by characterizing key rhizosphere metabolites such as flavonoids, siderophores, and organic acids that mediate beneficial plant–microbe communication (Sahoo et al., 2025). Integrating multi-omics data allows the construction of functional microbial networks that can be rationally engineered to sustain soil fertility and plant performance under adverse conditions (Thingujam et al., 2025).

### 3.7 Integrated pathways for climate-resilient soybean farming

**FIGURE 8:**
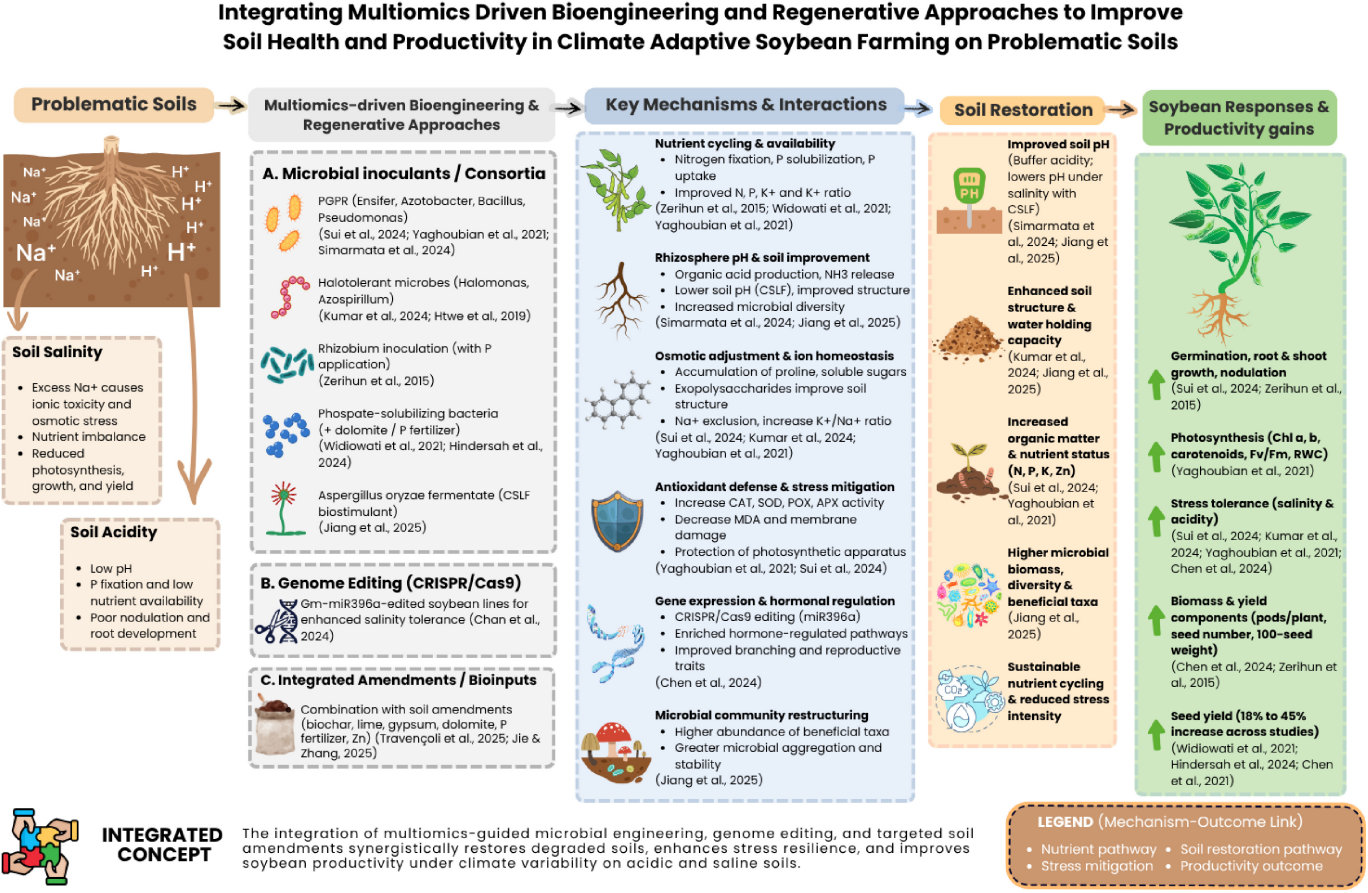
Conceptual framework for integrating multi-omics driven bioengineering and regenerative agronomy in soybean farming.

Combining omics-informed microbial bioengineering with eco-friendly bioameliorants such as biochar, compost, and microbial-enriched organic matter creates a synergistic pathway for soil health restoration (Figure 8). Biochar and compost improve soil buffering capacity, enhance cation exchange, and increase microbial habitat stability (Das and Pandey, 2025). When co-applied with microbial inoculants, they amplify root colonization, nutrient cycling, and carbon sequestration, ultimately improving soil–plant resilience against acid–saline stress (Gao et al., 2025).

Integrated farming systems, including crop rotation, intercropping, and cover cropping enhance soil structure, nutrient availability, and microclimatic conditions, thereby strengthening soybean resilience to climate variability (da Silva et al., 2024). Combining microbial bioengineering with these agronomic practices creates synergistic effects, optimizing resource use, reducing chemical inputs, and supporting sustainable, climate-resilient soybean production.

This integrated approach aligns with the principles of climate-smart agriculture, emphasizing biological intensification rather than chemical dependency (Kabato et al., 2025). It ensures productivity gains while restoring soil biodiversity, organic matter, and functional microbial networks. The adoption of such bioengineering frameworks can significantly reduce external inputs, enhance nitrogen fixation efficiency, and contribute to both food security and environmental sustainability (Jat et al., 2020).

### 3.8 Research gaps and future perspectives

Despite rapid progress, several knowledge gaps and operational challenges persist in applying multiomics-guided bioengineering to degraded soils:

1. Multiomics data integration: Most current studies analyze omics datasets in isolation. Integrative bioinformatics pipelines are needed to link genes, metabolites, and environmental responses (Raza et al., 2024; Roychowdhury et al., 2023).
2. Field-Level variability: SynComs validated under controlled conditions often fail in heterogeneous field environments due to microbial competition and environmental fluctuations (Jiang et al., 2023; Tariq et al., 2025).
3. Scalability and formulation: Developing stable, cost-effective microbial formulations that remain viable under field storage and transport remains a major bottleneck (Fadiji et al., 2024).
4. Policy and farmer adoption: Limited regulatory frameworks and awareness impede widespread adoption of engineered biological inputs in smallholder systems (Delgado-Baquerizo et al., 2025).

Addressing these gaps requires integrating systems biology, synthetic ecology, and agroecological design principles to co-create resilient, adaptable microbial solutions. The next frontier lies in coupling AI-assisted omics analytics and soil metamodeling to predict microbial behavior and optimize inoculant combinations for specific stress environments.

### 3.9 Synthesis and implications

The synergy between multiomics-driven rhizobiome bioengineering and bioameliorant technology represents a transformative framework for soil health restoration and climate-resilient soybean farming. This dual approach bridges molecular-level understanding with applied soil management, offering scalable pathways toward regenerative agriculture (Clagnan et al., 2024; Jing et al., 2022; Yusuf et al., 2025)

By decoding plant–microbe–soil signaling through multiomics and embedding beneficial microbes in supportive bioameliorant matrices, it is now possible to reconstruct degraded rhizospheres into biologically active, self-regulating ecosystems. The result is a sustainable soil–plant–microbe continuum capable of maintaining productivity, resilience, and ecosystem services under increasing environmental stress (Clagnan et al., 2024; Jing et al., 2022).

Despite remarkable progress, several gaps persist:

1. Multiomics data integration remains technically challenging due to variability in experimental conditions, sequencing platforms, and analytical workflows.
2. Scalability of SynCom applications requires validation under field heterogeneity and socio-economic constraints.
3. Long-term soil microbiome monitoring is essential to evaluate the sustainability and ecological trade-offs of engineered microbial interventions.
4. Cross-disciplinary collaboration among microbiologists, soil scientists, and data scientists is needed to advance predictive rhizosphere bioengineering.

A promising avenue involves AI-assisted omics integration, leveraging machine learning algorithms to correlate microbial gene networks with environmental metadata and plant performance metrics (Wu and Xie, 2025).

## 4 Conclusion

These findings highlight and demonstrate that integrating multiomics-driven bioengineering with regenerative approaches provides a robust, quantitative pathway to overcome soil acidity and salinity as key constraints to climate-adaptive soybean production on problematic soil. Advances in rhizobiome engineering, including synthetic microbial consortia, optimized bioinoculants, nutrient amendments, and CRISPR/Cas9-based gene editing, enable precise regulation of plant–microbe–soil interactions, enhancing nutrient bioavailability, ionic homeostasis, and stress tolerance. Collectively, these studies indicate that integrated approaches can enhance soybean productivity by 15%–45%, improved nitrogen-use efficiency by 30%, and achieved grain yields up to 3,228 kg ha^–1^. When combined with regenerative practices such as biochar, compost, and green manure, soil pH increased by 0.5–1.0 units, soil organic carbon by up to 40%, and soil biota abundance by 50%. Multiomics tools further strengthened these outcomes by enabling targeted, mechanism-based interventions. This review integrates multiomics and regenerative strategies within a unified framework for climate-resilient soybean systems. However, its conclusions are constrained by the limited number of studies, variability among datasets, and insufficient long-term field validation. In addition, the scalability of multiomics-based approaches is challenged by high costs, technical complexity, and limited accessibility, particularly in smallholder systems. Future research should prioritize large-scale field trials, scalable microbial technologies, and integrative multiomics modeling to support broader and practical adoption.

## Data Availability

Publicly available datasets were analyzed in this study. This data can be found here: no new datasets were created or deposited in public repositories.
